# Spontaneous Intraparenchymal Hepatic Hemorrhage as a Sequela of COVID-19

**DOI:** 10.7759/cureus.10447

**Published:** 2020-09-14

**Authors:** Saravgunjit Singh Daid, Adderly D Toribio, Seetha Lakshmanan, Ayad Sadda, Alan Epstein

**Affiliations:** 1 Internal Medicine, Roger Williams Medical Center, Providence, USA; 2 Gastroenterology and Hepatology, Roger Williams Medical Center, Providence, USA

**Keywords:** covid 19, intraparenchymal liver hemorrhage

## Abstract

Patients with coronavirus disease 2019 (COVID-19) have been presenting with varying signs and symptoms. The medical community is being updated with new knowledge about this disease daily. We present a case of intrahepatic hemorrhage in a patient diagnosed with C0VID-19 which we believe was caused by endothelialitis, which is a characteristic feature of COVID-19. Nevertheless, further continued studies are required to validate this point. We aim to educate the medical community about the possible complications by COVID-19 in the liver and highlight that N-acetylcysteine (NAC) may be a useful option in these cases.

## Introduction

Coronavirus disease 2019 (COVID-19) has been reported to have varying extrapulmonary presentations and complications. Early reports describe high venous thromboembolism (VTE) and disseminated intravascular coagulation (DIC) rates, but data are limited [1]. There have been reports of severe hemorrhage in a patient with COVID-19 but the origin and mechanism are unclear [2]. The reported incidence of major spontaneous hemorrhage in general admissions who are on prophylactic anticoagulation is 1% [3] but Palumbo et al. observed that rate to be 1.8% in their COVID-19 patient population [2]. Here we present a case of a 43-year-old female with a known COVID-19 infection developing acute transaminitis and intraparenchymal hepatic hemorrhage likely due to endothelialitis associated with COVID-19. 

## Case presentation

**Figure 1:**
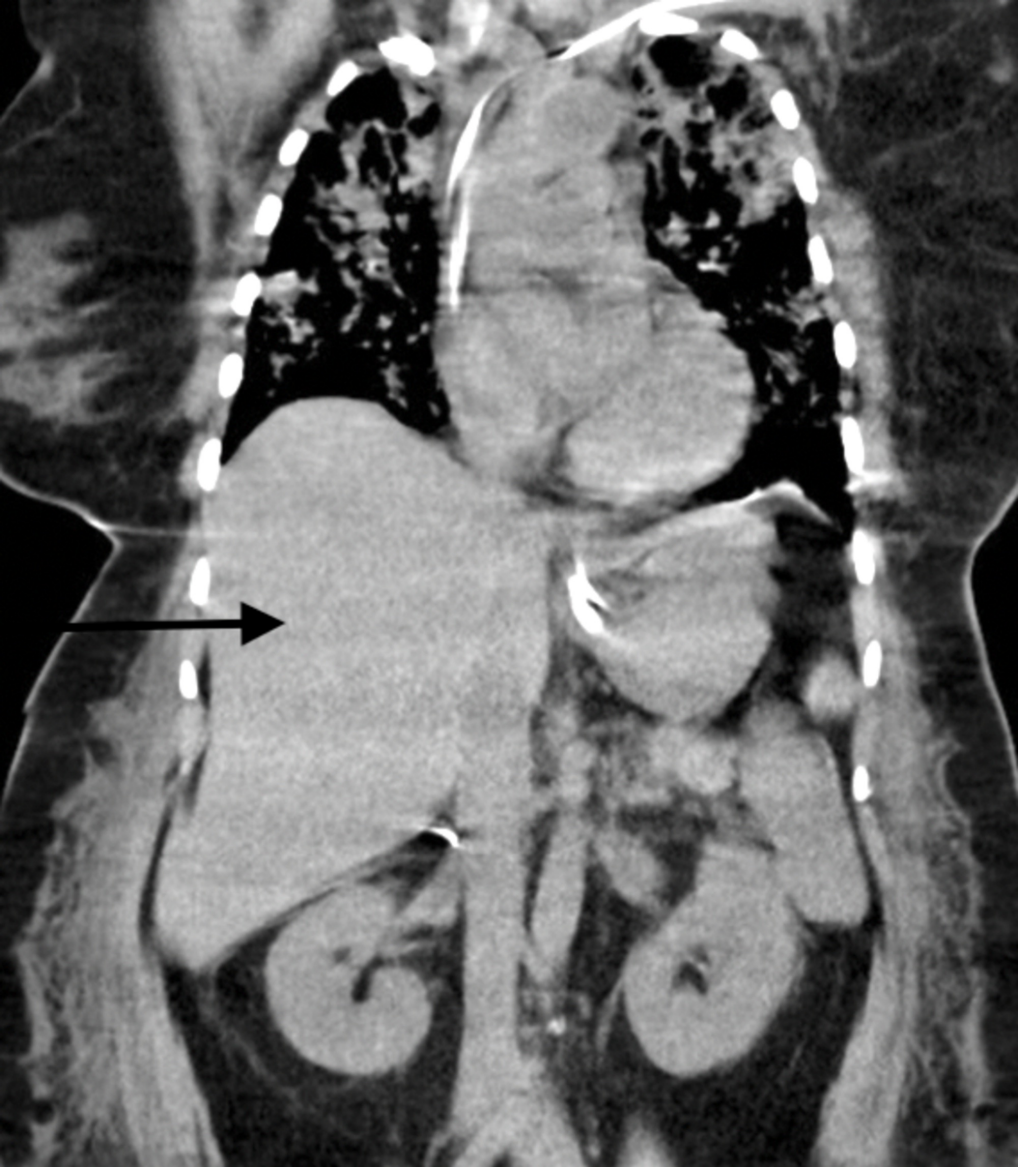
Non-contrast CT scan showing normal appearance of liver on the 8th day of hospitalization

A 43-year-old female with a past medical history of insulin-dependent diabetes mellitus, hypertension, and hyperlipidemia presented to the hospital with progressive shortness of breath, polyuria, dysuria, and polydipsia. The patient was diagnosed with COVID-19 by polymerase chain reaction (PCR) five days before coming to the hospital. Physical examination revealed hypoxia with decreased lung sounds and negative abdominal findings. Her labs on initial presentation revealed white blood count 21.2x109/L (normal range = 4.5 to 11.0 x 10^9/L), hemoglobin 15.1 gm/dl (normal range = 12 to 15.5 gm/dl), prothrombin time 12 seconds (normal range = 11 to 12.5 seconds), international normalized ratio (INR) 1.2 (normal range = < 1.1), fibrinogen 620 mg/dl (normal range = 200 to 400 mg/dL), D-dimer 2.03 mcg/mL fibrinogen equivalent units (FEU) (normal range < 0.5 mcg/mL FEU), blood glucose 430 mg/dl (normal range < 140 mg/dL) with an elevated anion gap, lactate 3.4 mmol/L (normal range = 0.5 to 1 mmol/L), and normal total bilirubin/liver enzymes. CT chest revealed extensive consolidative airspace opacities with ground glass opacities while the CT abdomen was normal (Figure 1). She was admitted to the intensive care unit (ICU) for diabetic ketoacidosis and progressive respiratory failure requiring endotracheal intubation on day 2 of hospitalization. Her pulmonary condition was further complicated by pulmonary edema likely related to cytokine release syndrome. She was initially treated with hydroxychloroquine and as her inflammatory markers continued to rise, she was administered tocilizumab on the day of intubation. Two days after the tocilizumab, the patient also received COVID-19 convalescent plasma. Her ICU course was further prolonged by superimposed bacterial infections including methicillin-sensitive Staphylococcus aureus pneumonia, treated with vancomycin, aztreonam, and meropenem, as well as fungal urinary tract infection, treated with fluconazole. She was successfully extubated eleven days later and was transitioned to the step-down unit.

**Figure 2:**
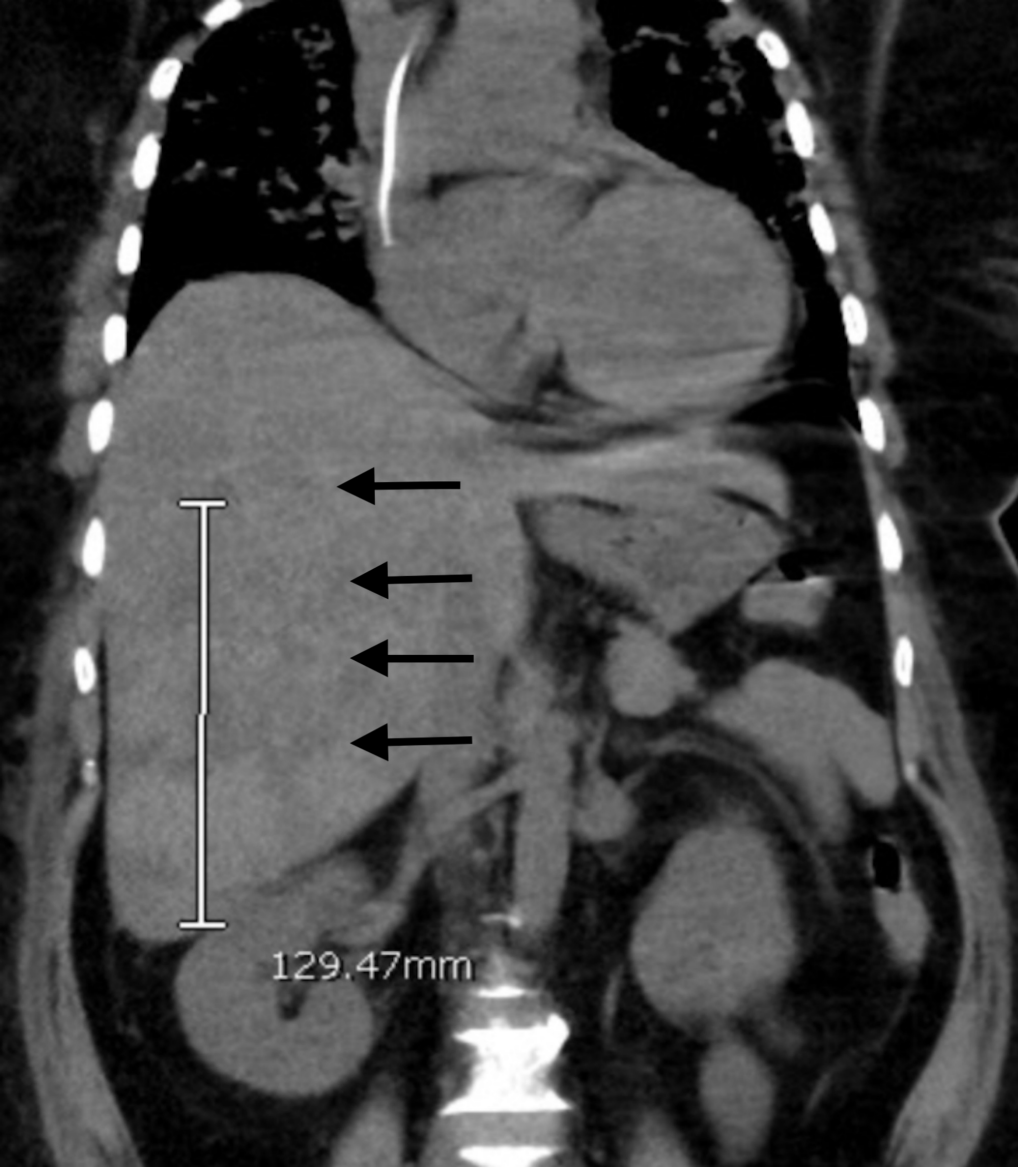
Non-contrast CT scan of the abdomen and pelvis showing heterogeneous area in the right side of the liver on the 15th day of hospitalization

**Figure 3:**
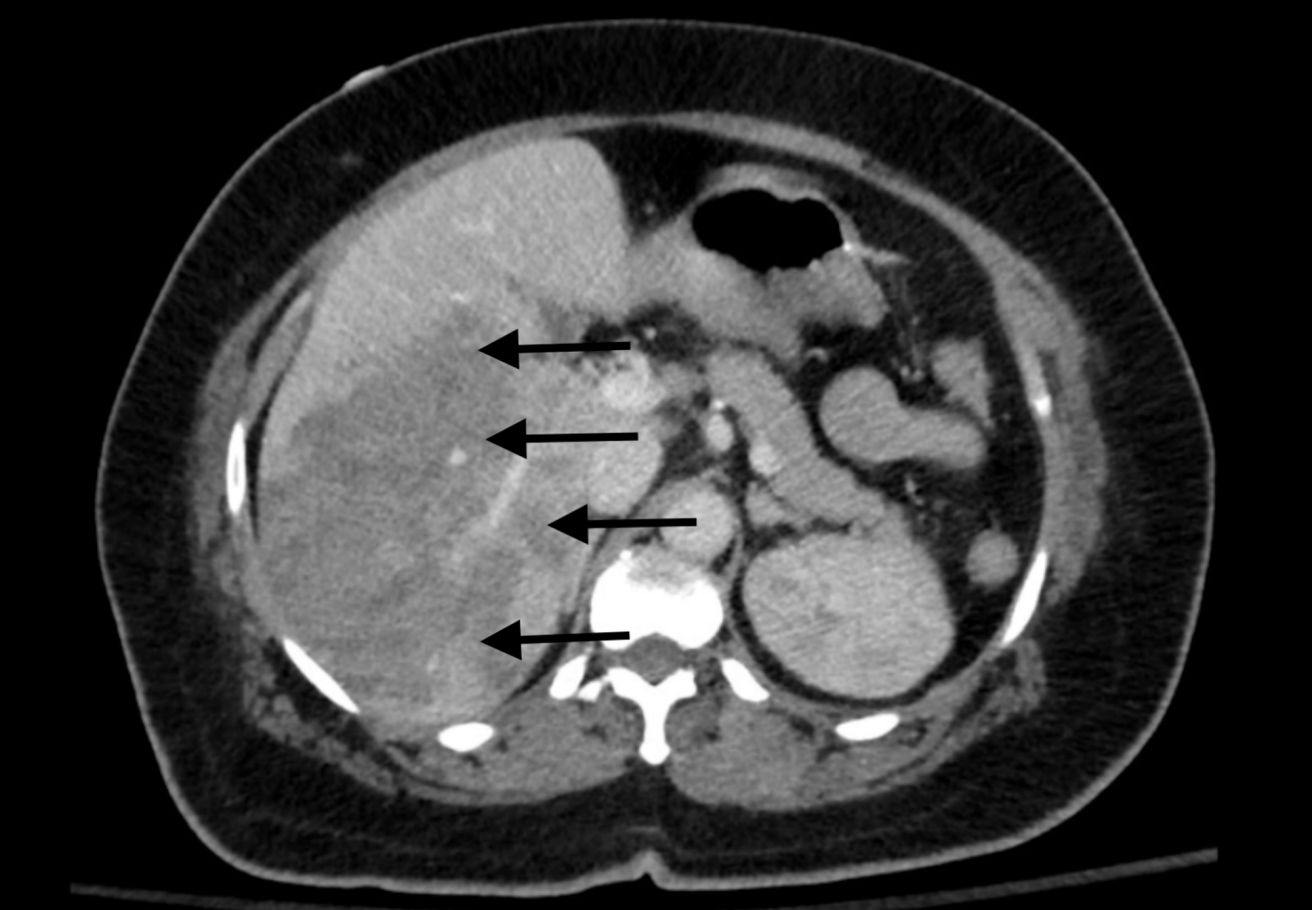
Three-phase liver CT confirming the heterogeneous area to be intra-parenchymal hemorrhage in the right side of the liver

On day 15 of admission, the patient developed severe right upper quadrant pain and back pain.Her complete blood count revealed hemoglobin of 6.1 gm/dl (normal range = 12 to 15.5 gm/dl), platelet count of 217,000/microliter of blood (normal range = 150,000 to 400,00/microliter of blood). The coagulation panel showed a prothrombin time of 13 seconds (normal range = 11 to 12.5 seconds) and an INR of 1.3 (normal < 1.1). Comprehensive metabolic panel revealed a sudden increase in liver enzymes with aspartate aminotransferase (AST) of 1111U/L (normal range = 5 to 40 U/L), alanine aminotransferase (ALT) of 1020 U/L (normal range = 19 to 25 U/L), and alkaline phosphatase (ALP) of 203 IU/L (normal range = 44 to 147 IU/L) with normal total bilirubin (normal range = 0.1 to 1. 2 mg/dL). CT abdomen without contrast revealed a heterogeneous attenuation pattern area in the right hepatic lobe (Figure 2). A follow-up ultrasound abdomen with Doppler confirmed patent vasculature. We obtained a three-phase liver CT with contrast which showed large areas of heterogeneous hypodensity and heterogenous enhancement most consistent with intraparenchymal hemorrhage (Figure 3). At this point, the patient's hemoglobin had also dropped by 2 gm/dl but fecal occult blood was negative, ruling out any gastrointestinal lumen bleeding. The patient was transfused with two units of packed red blood cells and her prophylactic anticoagulation was held.

While admitted, the patient’s AST peaked at 2177 U/L, ALT peaked at 1881 U/L and ALP peaked at 369 U/L. After discussing with the gastroenterology team, we started her on a trial of N-acetylcysteine (NAC) for her acute liver injury. Labs after a 21-hour infusion of NAC revealed a drastic and rapid improvement of her transaminitis. CT abdomen was repeated the next day which showed stabilization in the heterogeneous area in the right hepatic lobe. The patient continued to improve from the cardiorespiratory and gastrointestinal point of view and was discharged to an acute care rehab facility with normalized liver enzymes.

## Discussion

COVID-19 has been shown to use the angiotensin-converting enzyme (ACE) 2 receptors for cell entry [4]. These ACE2 receptors are highly expressed in lung alveolar type 2 cells and epithelial cells of the gastrointestinal system [4,5]. A recent epidemiologic study revealed that 43 out of 99 initial patients infected with COVID-19 had various degrees of liver function abnormality, with one patient having severe liver function damage. The patient had ALT of 7590U/L and AST of 1445U/L [6]. However, none have reported intraparenchymal hepatic hemorrhage associated with COVID-19.

This novel virus has been shown to cause hemorrhagic complications. Poyiadji et al. described a case of acute hemorrhagic necrotizing encephalopathy in a patient with COVID-19 [7]. Another study that looked at the lungs of patients with severe acute respiratory syndrome coronavirus-2 (SARS-CoV-2) infection at autopsy, noted structurally deformed capillaries and perivascular inflammation contributing to endothelial injury evidenced by endothelial cells showing disruption of intercellular junctions and basal membrane. The lungs of these patients also had widespread vascular thrombosis with microangiopathy and occlusion of alveolar capillaries [8]. Varga et al. also demonstrated the involvement of endothelial cells across vascular beds of different organs in a series of patients with COVID-19 [9]. In this study, one patient had lymphocytic endothelialitis in the lung, heart, kidney, and liver as well as liver cell necrosis on post mortem histology [9].

Tocilizumab has been associated with mild, short-lived transaminitis with antithrombotic and fibrinolytic effects through its action on factor XIII, chemerin, and plasminogen activator inhibitor 1 [10,11]. To our knowledge, there is no current evidence in the literature suggesting that tocilizumab may induce intrahepatic hemorrhage. As our patient received tocilizumab more than 10 days before, we believe her intrahepatic hemorrhage is less likely drug-related.

NAC is regularly used as an antidote in acute liver injury secondary to acetaminophen overdose (>4 grams per day in adults). NAC has been used in other non-acetaminophen related acute liver injuries, namely acute viral hepatitis or other drug toxicities. On top of the anti-inflammatory and antioxidant effects of NAC, it is also thought to improve oxygenation via vasodilation of microcirculatory blood flow to vital organs. The use of NAC has shown mortality benefit and shorter hospitalization duration, [12] making us more inclined to try it as a last resort in our patient. There have been many reports of successful NAC use in atypical cases including sickle cell crisis, ischemic hepatitis in cirrhotics, and septic shock.

## Conclusions

COVID-19 has presented with the involvement of multiple organ systems. It has shown a high affinity towards ACE2 receptors for cell entry. In our case presentation, the patient did not have any prior lesions in the liver on imaging and her liver function tests (LFTs) were also trending down after she came to floors from the ICU. But she did develop intraparenchymal liver hemorrhage which we believe might be due to endothelial injury caused by COVID-19, although we have not found any other cases showing the same findings. We want to educate, by means of this case report, that if a patient with COVID-19 complains of right upper quadrant pain and increased LFTs then spontaneous intraparenchymal hemorrhage should be in the differential diagnosis. 
